# Supplementary material for: VQ‐Wave: A Physics‐Driven Spatiotemporal Deep Learning Approach for Noncontrast‐Enhanced Lung Ventilation and Perfusion MRI
Source: Magn Reson Med. 2026 May 10;96(3):1427–42. doi: 10.1002/mrm.70423 (PMC13327449; doi:10.1002/mrm.70423)
Supplement: Supplementary file 1 — Table S1: Physiological parameters, signal characteristics, and training goals of the synthetic data generator. The assigned probabilities reflect the sampling frequency of the center voxel's tissue class during stochastic training. Parameter bounds, pathological defect rates, and dynamic noise profiles were explicitly defined to force the network to generalize across diverse physiological conditions and bridge the clinical domain gap. Table S2: Summary of network optimization and training hyperparameters. The table details the specific machine learning configurations, including the optimization schedule, loss function definitions, temporal acquisition boundaries, and the spatial augmentation strategies. Figure S1: Visual comparison of reconstruction stability in a healthy volunteer. Comparison of fractional ventilation (left) and perfusion (right) maps reconstructed using a standard long acquisition (N = 140, top row) versus an accelerated short acquisition (N = 40, bottom row). VQ‐Wave and the matrix pencil (MP) reference produce high‐quality functional maps from the full time‐series. When the acquisition is reduced to 15 s (N = 40), VQ‐Wave maintains diagnostic image quality with no loss of structural detail. In contrast, the MP perfusion map exhibits visible degradation, including signal dropout in the lower lobes and increased background noise, indicating a failure to resolve the cardiac signal from the limited data. [file MRM-96-1427-s001.pdf]

Tissue Class / Feature	Signal characteristics	Parameter Ranges / Probabilities	Training goal
**Lung parenchyma**	Low to moderate baseline, variable A_v and A_q	**Probability:** 40%<br><br>**Baseline:** 15–250 a.u.	Learning simultaneous ventilation and perfusion separation
**Pulmonary vessels**	High baseline, strong A_q, $A_v \approx 0$. Includes “weak vessel” variants to improve sensitivity	**Probability:** 30%<br><br>**Baseline:** 200–800 a.u.	Learning that high signal intensity does not imply ventilation; identifying pure perfusion sources
**Static tissue**	High baseline, zero dynamic amplitude ($A_v = A_q = 0$)	**Probability:** 20%<br><br>**Baseline:** 300–800 a.u.	Teaches the network to predict zero-amplitude in static regions (e.g. muscle, fat)
**Air background**	Very low baseline signal and absence of any V/Q modulation ($A_v = A_q = 0$)	**Probability:** 10%<br><br>**Baseline:** 3–20 a.u.	Ensures robustness against low-SNR / random phase noise
**Physiological frequencies**	Shared underlying respiratory and cardiac rates applied across the entire field of view	**Ventilation (f_v):** 0.10–0.60 Hz<br><br>**Perfusion (f_q):** 0.75–2.0 Hz	Trains the network to track temporal dynamics independently of local tissue amplitudes
**Pathological defects**	Regional absence of A_v or A_q modulation within otherwise standard lung parenchyma	**Ventilation Defect:** 10% probability<br><br>**Perfusion Defect:** 10% probability	Force the network to correctly identify physiological voids (e.g., embolisms, air trapping)
**Irregular dynamics & noise**	Non-stationary baseline drift, erratic amplitude spikes, and noise	**Baseline Drift:** Additive Gaussian random walk.<br><br>**Sighs/Spikes:** up to 50% amplitude increase<br><br>**Amplitude Jitter:** Random heart beat-to-beat variance<br><br>**Frequency Drift:** Non-stationary variation in f_v and f_q<br><br>**Noise (σ):** 1.0–17.5	Ensure temporal filter stability against patient motion, deep sighs, heart rate variability, shifting respiratory rates or baseline drifts

Supporting Information Table S1. Physiological parameters, signal characteristics, and training goals of the synthetic data generator**.** Note: The assigned probabilities reflect the sampling frequency of the center voxel’s tissue class during stochastic training. Parameter bounds, pathological defect rates, and dynamic noise profiles were explicitly defined to force the network to generalize across diverse physiological conditions and bridge the clinical domain gap.

Category	Parameter	Value / Setting
**Optimization**	Optimizer	AdamW
	Base learning rate	1×10^{-3}
	Weight decay	1×10^{-4}
	Number of samples	10^6 spatial patches
	Training schedule	50 epochs
	Batch size	1024 spatial patches
	Learning rate scheduler	Cosine annealing warm restarts (T_0=10, T_{mult}=2)
**Loss functions**	Amplitude targets (A_v, A_q)	Smooth L1 loss
	Frequency & phase targets (f_v, f_q, ϕ_v, ϕ_q)	Mean squared error (MSE)
**Acquisition bounds**	Temporal resolution	Uniformly sampled in [0.20, 0.33] seconds
	Sequence length	Uniformly sampled [40, 190] frames
**Spatial augmentation**	Local 3×3 patch rotation	Random 90° rotations ($k \in [0,1,2,3]$)
	Local 3×3 patch flipping	Horizontal and Vertical flips (p = 0.5)

Supporting Information Table S2. Summary of network optimization and training hyperparameters. The table details the specific machine learning configurations, including the optimization schedule, loss function definitions, temporal acquisition boundaries, and the spatial augmentation strategies

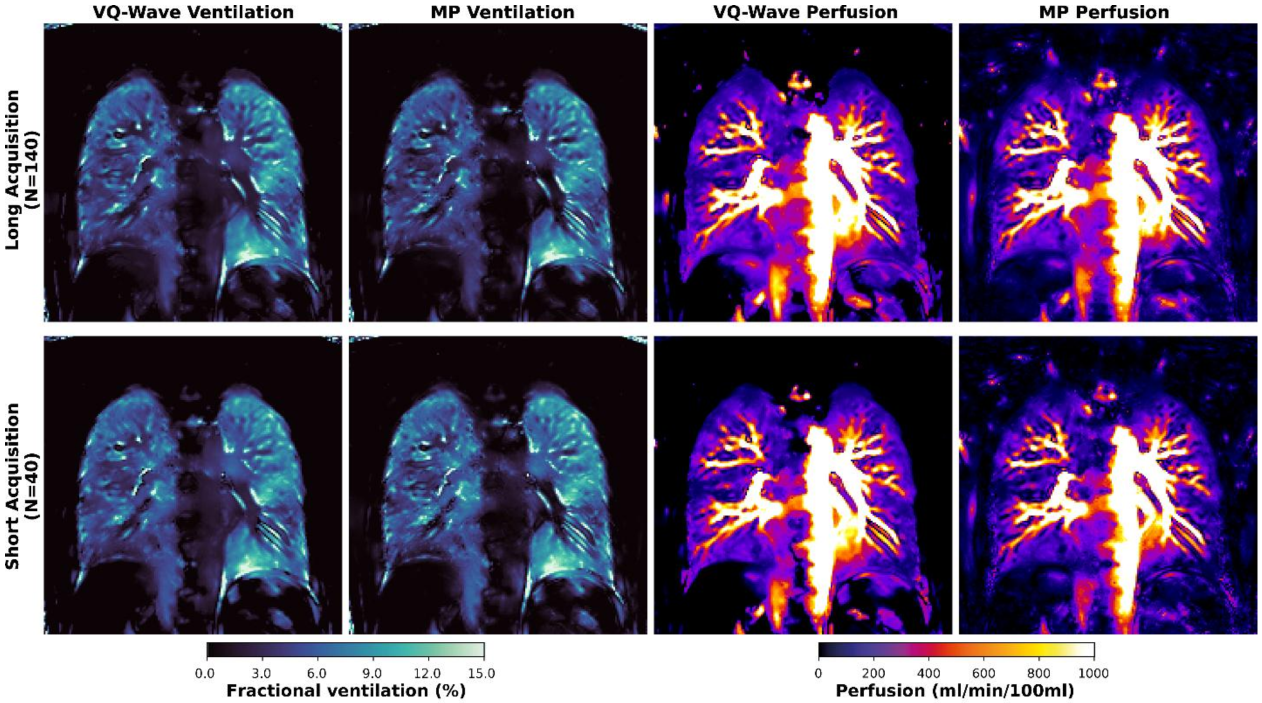

Supporting Information Figure S1. Visual comparison of reconstruction stability in a healthy volunteer. Comparison of fractional ventilation (left) and perfusion (right) maps reconstructed using a standard long acquisition (N=140, top row) versus an accelerated short acquisition (N=40, bottom row). VQ-Wave and the matrix pencil (MP) reference produce high-quality functional maps from the full time-series. When the acquisition is reduced to 15 seconds (N=40), VQ-Wave maintains diagnostic image quality with no loss of structural detail. In contrast, the MP perfusion map exhibits visible degradation, including signal dropout in the lower lobes and increased background noise, indicating a failure to resolve the cardiac signal from the limited data.
